# Association Between Scalp High-Frequency Oscillations and Burden of Amplitudes and Epileptiform Discharges (BASED) Scores in Infantile Epileptic Spasms Syndrome

**DOI:** 10.3390/biom15050697

**Published:** 2025-05-10

**Authors:** Keisuke Maeda, Himari Tsuboi, Nami Hosoda, Junichi Fukumoto, Shiho Fujita, Shunta Yamaguchi, Naohiro Ichino, Keisuke Osakabe, Keiko Sugimoto, Gen Furukawa, Naoko Ishihara

**Affiliations:** 1 Department of Clinical Physiology, Fujita Health University School of Medical Sciences, 1-98 Dengakugakubo, Kutsukake-cho, Toyoake 470-1192, Japan; 2 Department of Clinical Laboratory, Fujita Health University Hospital, 1-98 Dengakugakubo, Kutsukake-cho, Toyoake 470-1192, Japan; 3 Department of Medical Sciences Education, Fujita Health University School of Medical Sciences, 1-98 Dengakugakubo, Kutsukake-cho, Toyoake 470-1192, Japan; 4 Department of Pediatrics, Fujita Health University School of Medicine, 1-98 Dengakugakubo, Kutsukake-cho, Toyoake 470-1192, Japan

**Keywords:** infantile epileptic spasms syndrome, high-frequency oscillation, burden of amplitudes and epileptiform discharges, west syndrome, epilepsy, electroencephalography

## Abstract

Tools for measuring the likelihood of relapse in infantile epileptic spasms syndrome (IESS) treatment could aid clinicians in making critical management decisions. High-frequency oscillations (HFOs), transient bursts of electroencephalography (EEG) activity with frequencies beyond 80 Hz, are a new and promising noninvasive biomarker. The present study aimed to investigate the association between the Burden of Amplitudes and Epileptiform Discharges (BASED) scores, an interictal EEG grading scale for IESS, and scalp HFOs in patients with IESS. The study enrolled 50 patients, 25 with a clinical diagnosis of IESS and 25 without epilepsy. The percentage of patients with at least one scalp HFO detected, stratified by BASED scores, differed significantly: for BASED scores ≤ 2, 7.7%; for 3, 16.7%; for 4, 87.5%; and for 5, 100% (*p* < 0.001). Compared with BASED scores ≤ 2, the median scalp HFO detection rate was significantly highest for BASED scores of 5 (median [IQR]: 6.24 [2.25–8.32], *p* < 0.001), followed by BASED scores of 4. The scalp HFO detection rates showed a better performance in estimating patients with BASED scores of 4 and 5. It is hoped that scalp HFOs can be used as an objective indicator to validate the results of BASED scores.

## 1. Introduction

Infantile epileptic spasms syndrome (IESS) is a severe form of epileptic encephalopathy characterized by the onset of epileptic spasms in infants [1,2,3]. The syndrome includes West syndrome, where patients have a triad of epileptic spasms, hypsarrhythmia on electroencephalography (EEG), and developmental plateauing or regression [4,5,6]. The ideal outcome after successful IESS treatment is the resolution of clinical spasms and normalization of the EEG; however, this is not always obtainable. Treating IESS is a complex and difficult process, and the best treatment depends on the needs of the patient [7]. Therefore, tools to predict the initial drug response to IESS treatment and to measure the likelihood of relapse could aid clinicians in making critical management decisions.

The Burden of AmplitudeS and Epileptiform Discharges (BASED) score, initially reported in 2015 [8] and revised in 2021 [9], is an interictal EEG grading scale for pediatric patients with IESS. The BASED score can be a useful tool in diagnosing IESS, assessing the treatment response, and predicting prognosis [10,11]. Although the BASED score has shown excellent inter-rater reliability (IRR) [9,12], relying solely on a manual labor and human vision to quantify EEG data remains subjective and difficult. Therefore, more objective and accurate interictal EEG features are needed for IESS treatment.

High-frequency oscillations (HFOs) measured with scalp EEG (scalp HFOs) have shown promise as noninvasive biomarkers of drug treatment and pediatric epilepsy severity [13,14,15,16]. HFOs are defined as transient spontaneous low-amplitude oscillations at a high frequency (≥80 Hz) standing out of the background EEG signal [17,18]. Several studies have shown that scalp HFOs are associated with the localization of epileptogenic activity in IESS [19,20,21]. Scalp HFOs have also been reported to be associated with spasm and treatment effectiveness in West syndrome [17,22]. However, to our knowledge, no studies have found a relationship between the 2021 BASED scores and scalp HFOs. Although visual validation is required, scalp HFO detection can be assessed semi-automatically and objectively [23,24]. By clarifying the relationship between the 2021 BASED scores and scalp HFOs, it is hoped that scalp HFOs can be used as an objective indicator to validate the results of BASED scores.

Given this background, the present study aimed to investigate the association between the 2021 BASED score and scalp HFOs recorded by scalp EEG in patients with IESS. The findings could be expected to help promote scalp HFOs as an adjunctive tool in the diagnosis and therapeutic management of IESS.

## 2. Materials and Methods

### 2.1. Study Participants

This study enrolled 25 patients (13 male) who had received a clinical diagnosis of IESS by a child neurologist at Fujita Health University Hospital between 2018 and 2023. We selected one scalp EEG recording that met the following inclusion criteria from each patient: (1) recorded at a high sampling frequency (≥1000 Hz); (2) recorded during intermittent seizures; and (3) the recordings included sufficient non-rapid eye movement sleep. We also included an equal number of age-matched controls (*n* = 25), consisting of patients with no known neurological disorders who had undergone an EEG because of suspected epilepsy, which was subsequently ruled out. Consequently, scalp EEG recordings from 50 patients (25 patients with IESS and 25 patients without epilepsy) were included in the final analysis.

This study adhered to the principles laid out in the Declaration of Helsinki and subsequent amendments [25] and was approved by the Ethics Committee of Fujita Health University (Approval No. HM22–143). Patient consent was acquired using an opt-out method on the university website.

### 2.2. Scalp EEG Recording and Data Selection

Scalp EEG recordings were obtained using the Neurofax system (Nihon-Kohden, Tokyo, Japan), utilizing 23 electrodes placed according to the international 10–20 system and a digital sampling frequency of 1000 Hz. The analysis was performed in each of the following channels in an average montage: Fp1, Fp2, F3, F4, C3, C4, P3, P4, O1, O2, F7, F8, T3, T4, T5, T6, Fz, Cz, and Pz. An artifact-free approximately 600 s of non-REM sleep EEG was selected by a specialist technician certified by the Japanese Society of Clinical Neurophysiology who was blinded to the clinical information. After the EEG data were covered in European Data Format, scalp HFO detection was performed.

### 2.3. Detection of Scalp HFOs

In this study, scalp HFOs were defined as oscillatory events with at least four oscillations of sinusoidal-like morphology and a center frequency occurring between 80 and 250 Hz that stood out from the background signal [26]. We constructed the 19 channels using an average montage and performed scalp HFO detection in all channels. Scalp HFOs were first detected automatically using an automated HFO detector, and then the auto-detected HFOs were verified by visual inspection by experienced reviewers. The auto-detected HFOs were performed using the Hilbert Detector proposed by Crépon et al. [27] which has been clinically validated. The Hilbert Detector operates in two stages. In the first stage, the band-pass filtering of the EEG signals is performed to limit the frequency content of each signal to the frequency band of interest. The signal envelope is then computed using the Hilbert transform. The second stage, its local maximum (corresponding to the estimated scalp HFO events) are automatically detected using a threshold set to five times the standard deviation (SD) of the envelope calculated over the entire EEG recording. The auto-detected HFOs were then validated through visual inspection, and oscillations that appeared to be contaminating noise or muscle activity were excluded. Visual verification of the auto-detected HFOs was performed by a specialist technician certified by the Japanese Society of Clinical Neurophysiology. The scalp HFO detection rate (detections/min) was calculated by dividing the number of scalp HFO events detected in all channels by the duration of the EEG recordings analyzed. All analyses were computed using HFOApp [28] and MATLAB R2022a software (MathWorks, Natick, MA, USA).

### 2.4. BASED Score Counts

The BASED score is an interictal EEG grading scale that was reported in 2015 [8] and revised in 2021 by Mytinger et al. [9]. We graded the EEG recordings of all subjects according to the revised BASED scoring criteria [9]. BASED scores were calculated in random order by blinded reviewers who were specialist technicians certified by the Japanese Society of Clinical Neurophysiology. The reviewers assessed channels on the longitudinal bipolar montage (Fp1–F7, F7–T3, T3–T5, Fp1–F3, F3–C3, C3–P3, Fz–Cz, Cz–Pz, Fp2–F4, F4–C4, C4–P4, Fp2–F8, F8–T4, and T4–T6). The scale ranges from 0 (normal) to 5 (most epileptic), with a score of 3 to 5 applied to the most epileptic 5 min sleep epochs; if no score is reached, a score of 0 to 2 is applied according to the whole EEG data. The 2021 BASED scoring criteria are shown in the Supplementary Materials (Table S1).

### 2.5. Data Collection

IESS was diagnosed according to the 2017 International League Against Epilepsy definition by specialists in clinical epilepsy who were blinded to the HFO analysis [5,29,30]. Clinical data for each subject, including sex, age at EEG recording and IESS onset, and medication history, were collected using a chart review.

### 2.6. Statistical Analysis

Because the scalp HFO detection rates and analyzed EEG recording durations had log-normal distributions, we present these data as median values with interquartile ranges (IQRs). Other normally distributed continuous variables are presented as means and SDs. First, using a two-tailed chi-square test, the proportion of patients with scalp HFOs (categorical variable) was compared between patients with IESS and patients without epilepsy. Similarly, the scalp HFO detection rates (continuous variable) between patients with IESS and patients without epilepsy were compared using the Wilcoxon rank-sum test. In addition, patients with IESS were divided into the following two categories according to seizure control performance: patients with good seizure control who had no seizures within at least the previous 2 months, and patients with poor seizure control. Similar comparisons of scalp HFOs were performed between patients with IESS with good and poor seizure control and patients without epilepsy. Next, the proportion of patients with scalp HFOs was compared across BASED scores using a two-tailed chi-square test. Similarly, scalp HFO detection rates among BASED scores were compared using the Steel–Dwass multiple comparison test. Furthermore, logistic regression analysis was conducted to estimate odds ratios (ORs) and 95% confidence intervals (CIs) for HFO appearance according to BASED scores. Patients with scalp HFOs were also divided into the following two groups based on median HFO detection rates (1.46 detections/min): a high detection rate group and a low detection rate group. Similarly, logistic regression analyses were performed to examine the association between high scalp HFO detection rates and BASED scores. Finally, the optimal discriminability of each BASED score using the scalp HFO detection rate was evaluated by receiver operating characteristic (ROC) analysis, and the area under the ROC curve (AUC-ROC) was calculated to compare performance. *p*-values < 0.05 were considered statistically significant. All statistical analyses were performed using JMP software ver. 17 (SAS Institute, Inc., Cary, NC, USA).

## 3. Results

### 3.1. Basic Characteristics

Table 1 shows the basic characteristics of the study subjects stratified by IESS status. The total number of participants was 24 males and 26 females, including 13 males and 12 females with IESS. The mean ages (SDs) of the participants without epilepsy and with IESS at the time of the EEG recordings were 3.7 (4.6) and 4.5 (4.5) years, respectively. No significant differences in gender or age were found between patients without epilepsy and with IESS (*p* = 0.57 and 0.54, respectively).

### 3.2. Comparison of Scalp HFOs in Patients with IESS with Good and Poor Seizure Control and Patients Without Epilepsy

Table 2 shows the scalp HFO results for patients with IESS with good and poor seizure control and patients without epilepsy. Compared with patients without epilepsy, a significantly higher percentage of patients with IESS had at least one scalp HFO detected (8.0% vs. 72.0%, respectively; *p* < 0.001). The median scalp HFO detection rate was significantly higher in patients with IESS (median [IQR]: 1.04 [0.00–4.45]) than in patients without epilepsy (0.00 [0.00–0.00], *p* < 0.001).

The percentage of patients with at least one scalp HFO detected was highest among those with IESS with poor seizure control (83.3%), followed by those with good seizure control (42.9%), and lowest in patients without epilepsy (8.0%, *p* < 0.001). The median scalp HFO detection rate was significantly higher in patients with IESS with poor seizure control (median [IQR]: 1.77 [0.60–6.36]) than in those with good seizure control (0.00 [0.00–0.30], *p* < 0.01) and patients without epilepsy (0.00 [0.00–0.00], *p* < 0.001). The median scalp HFO detection rate was also significantly higher in patients with IESS with good seizure control than in patients without epilepsy (*p* < 0.05).

### 3.3. Comparison of Scalp HFOs Stratified by BASED Scores

**Figure 1:**
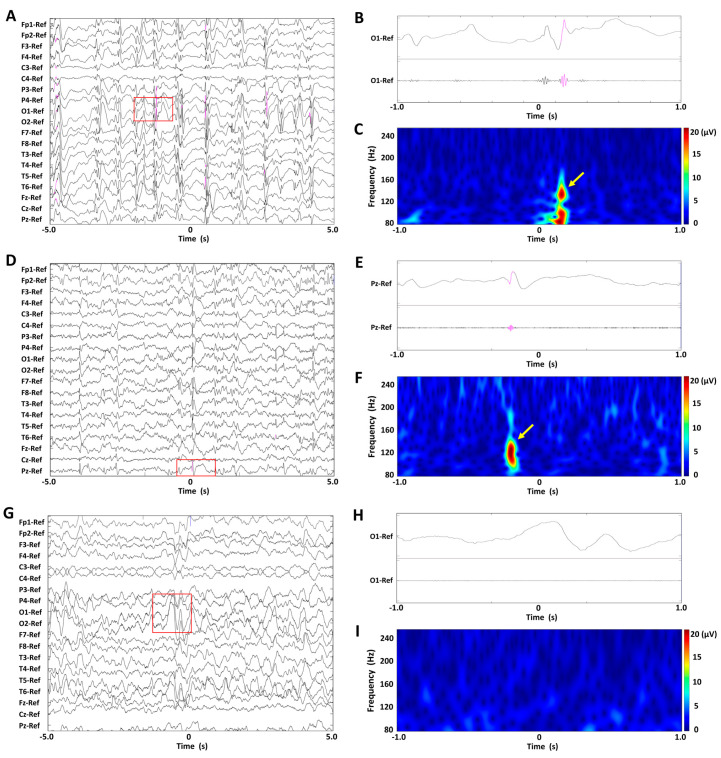
Findings of scalp HFOs and BASED scores in patients with infantile epileptic spasms syndrome. (**A**–**C**) The BASED score was 5. (**D**–**F**) The BASED score was 4. (**G**–**I**) The BASED score was 3. (**A**,**D**,**G**) Unfiltered EEG recordings with normal time scales. (**B**,**E**,**H**) Lead signals with extended time scales indicated by red rectangles and bandpass-filtered EEG recordings at 80–250 Hz. (**C**,**F**,**I**) Spectrograms of the EEG data in the time–frequency analysis for the EEG recordings are enclosed in red rectangles. Scalp HFOs are observed in the unfiltered and filtered EEG recordings (pink traces) and correspond to spectral blobs in the time–frequency analysis (yellow arrows).

Figure 1A,D,G show EEG recordings of representative BASED scores of 5, 4, and 3 in a patient, displayed under unfiltered display conditions with normal time scales. Figure 1B,E,H show unfiltered EEG recordings with extended time scales and band-pass-filtered EEG recordings at 80–250 Hz, for the EEG recordings enclosed in red rectangles. Figure 1C,F,I show spectrograms of the EEG data in a time–frequency analysis, for the EEG recordings enclosed in red rectangles. No scalp HFOs were found in the EEG recording of BASED score 3, whereas one scalp HFO was seen in the EEG recording of BASED score 4 and numerous scalp HFOs were seen in the EEG recording of BASED score 5.

**Figure 2:**
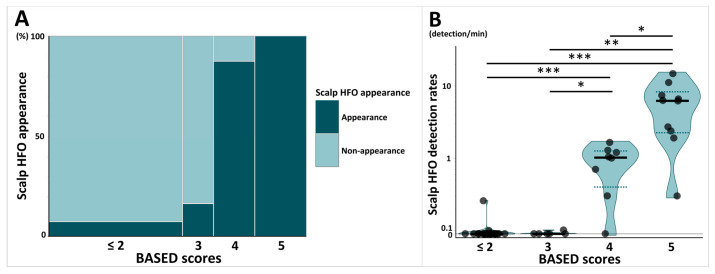
Comparison of scalp HFOs stratified by BASED scores. (**A**) Mosaic plot shows the proportion of patients with scalp HFO appearance stratified by BASED score. (**B**) Violin plot shows the comparison of scalp HFO detection rates stratified by BASED score. Subjects were classified into four groups stratified by BASED score: less than 2, 3, 4, and 5. Each point shows individuals, and each shaded area shows the distribution of the scalp HFO detection rates. The bold horizontal lines indicate the median values for each group, and the dotted lines indicate the quartile range (25th–75th percentiles). Abbreviations: BASED score, burden of amplitudes and epileptiform discharges score; HFO, high-frequency oscillation. * *p* < 0.05. ** *p* < 0.01. *** *p* < 0.001.

The mosaic plot in Figure 2A shows the proportion of patients with scalp HFOs stratified by BASED scores. The percentage of patients with at least one scalp HFO detected, stratified by BASED scores, differed significantly, ranging from 7.7% for BASED scores ≤ 2, 16.7% for BASED scores of 3, 87.5% for BASED scores of 4, and 100% for BASED scores of 5 (*p* < 0.001).

The violin plot in Figure 2B shows a comparison of scalp HFO detection rates stratified by BASED scores. The median scalp HFO detection rate for BASED scores of 5 (median [IQR]: 6.24 [2.25–8.32]) was significantly higher than those of BASED scores of 4 (1.02 [0.40–1.26], *p* = 0.02), 3 (0.00 [0.00–0.03], *p* = 0.006), and ≤2 (0.00 [0.00–0.00], *p* < 0.001). BASED scores of 4 were also associated with a significantly higher scalp HFO detection rate than BASED scores of 3 and ≤2 (*p* = 0.04 and *p* < 0.001, respectively).

### 3.4. ORs and 95% CIs of Scalp HFOs According to BASED Scores

Table 3 summarizes the association between scalp HFOs and BASED scores using BASED scores ≤ 2 as the reference. Statistical analyses were performed using logistic regression analysis. Compared with BASED scores ≤ 2, the OR for scalp HFOs was significantly highest for BASED scores of 5 (OR [95% CI] = 205.8 [18.0–29,736.5], *p* < 0.001), followed by BASED scores of 4 (49.0 [7.0–652.1], *p* < 0.001). The OR for a high scalp HFO detection rate was significantly higher for BASED scores of 5 than for BASED scores ≤ 2 (OR [95% CI] = 335.7 [25.0–52,662.8], *p* < 0.001). A 1-point increase in the BASED score was associated with an increased likelihood of a rise in the appearance of scalp HFOs (OR [95% CI] = 3.1 [1.9–5.9], *p* < 0.001) and a high scalp HFO detection rate (65.8 [6.5–2562.9], *p* = 0.004).

### 3.5. ROC Curves of Scalp HFO Detection Rates for Predicting BASED Scores

**Figure 3:**
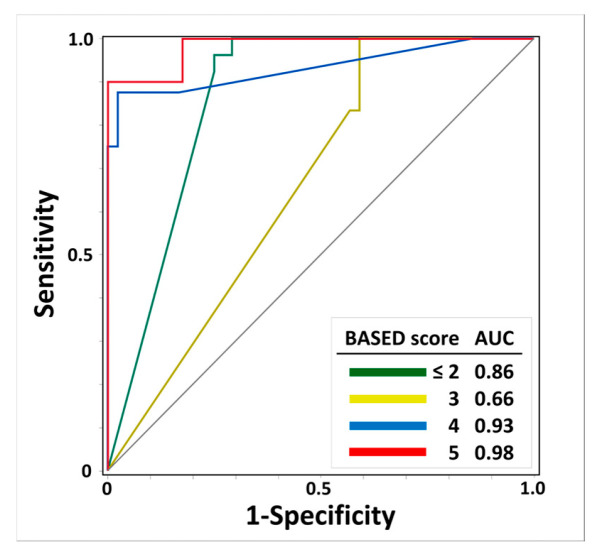
Receiver operating characteristic curves of scalp HFO detection rates for predicting BASED scores. The ROC curve and AUC of scalp HFO detection rates for predicting BASED score are shown. Abbreviations: AUC, area under the curve; BASED score, burden of amplitudes and epileptiform discharges score.

Figure 3 shows the results regarding the AUC-ROC and scalp HFO detection rates for the prediction of BASED scores. The AUC-ROCs of the scalp HFO detection rates for BASED scores ≤ 2, 3, 4, and 5 were 0.86, 0.66, 0.93, and 0.98, respectively, with the highest identification for a BASED score of 5, followed by a BASED score of 4.

## 4. Discussion

This study investigated the association between 2021 BASED scores and scalp HFOs recorded by scalp EEG in patients with IESS. The results showed that the proportion of patients with scalp HFOs was significantly highest for BASED scores of 5, followed by BASED scores of 4. In addition, scalp HFO detection rates were significantly highest for BASED scores of 5, followed by BASED scores of 4. By contrast, scalp HFOs were seldom detected in patients with BASED scores ≤ 3. Furthermore, an increased risk of a high scalp HFO detection rate was observed in patients with a BASED score of 5 compared with a BASED score ≤ 2. Finally, scalp HFO detection rates showed better performance in estimating patients with BASED scores of 4 and 5.

Several recent reviews have reported that scalp HFOs appear in various epilepsy syndromes [13,31]. Previous studies have reported that patients with IESS, including West syndrome, also present with scalp HFOs [32,33]. Similarly to previous studies, we also observed the occurrence of scalp HFOs in patients with IESS. When viewed in relation to epileptic spasms, HFOs have been observed in association with epileptic spasms [22]. The appearance of HFOs is associated with seizure frequency [34], and scalp HFOs are known to be detected more frequently in patients with epilepsy at high risk of seizure recurrence [35,36,37]. HFOs are implicated both before and during the onset of seizures, and the mechanism of HFO development appears to depend on the type of seizure onset pattern [38]. A better understanding of whether HFOs cause or are caused by seizures requires a deeper analysis of the mechanisms of generation of different kinds of HFOs. It should also be emphasized that in the present study, more scalp HFOs were found in patients with IESS with a higher seizure risk.

The BASED score has been used as an accurate, reliable, and feasible EEG grading scale for pediatric patients with IESS [11,39]. This scoring method has been presented as an alternative to hypsarrhythmia for assessing the treatment efficacy in pediatric patient with IESS [8,40]. HFOs have also been found to be associated with treatment responsiveness, as previous studies have reported that increased HFO detection rates following IESS treatment are highly suggestive of treatment non-response [41,42,43]. Although both HFOs and BASED scores are associated with treatment responsiveness, the relationship between BASED scores and HFOs has not been well studied. The present study demonstrated that few scalp HFOs were detected for BASED scores ≤ 3, whereas many scalp HFOs were detected for BASED scores of 4 and 5. A high frequency of scalp HFOs was observed, particularly for BASED scores of 5. A retrospective study reported that children with IESS accompanied by a BASED score > 3 after the initial response to adrenocorticotropic hormone therapy carry a high risk of relapse within 1 year [11]. Therefore, it is important to be able to determine reliably whether the BASED score is >3 when assessing the risk of relapse in patients with IESS.

A major strength of this study is that the scalp HFO detection rates showed better performance levels in estimating patients with BASED scores of 4 and 5. Hence, the present findings support the results of a previous study finding that increased HFO detection rates are a better predictor of the risk of active epileptic spasms than hypsarrhythmia alone [41]. Furthermore, in a retrospective study, electrographic remission was defined as pretreatment BASED scores of 4 or 5 improving to ≤3 [9]. Therefore, the present findings suggest that scalp HFOs may be an indicator of IESS remission in the future.

The BASED score has demonstrated superior IRR compared to the clinician evaluations of hypsarrhythmia [9,12], but cannot be entirely ruled out by subjective judgments. The detection of scalp HFOs has been shown to be quite feasible and timely for objective analysis [41,44]. Thus, scalp HFOs may be useful as an objective measure to validate BASED scores. However, the visual identification of scalp HFOs requires experienced experts, and IRR may not be optimal [45,46]. Thus, this could involve limitations similar to those for BASED scores. However, previous studies on the performance of automatic HFO analysis have reported that a predictive model without visual verification performed as well as a model with visual verification [41]. When using BASED scores to predict treatment response or a relapse of IESS, validation along with scalp HFO results may provide a higher IRR.

This study has some limitations. First, scalp HFOs were assessed using sleep EEG with relatively few artifacts. Therefore, we did not necessarily expect that we would obtain similar results from awake EEGs. In order to accurately evaluate scalp HFOs on awake EEGs, methods are needed to compensate for various EEG artifacts, including muscle, movements, etc. [47]. In the present study, we visually excluded “false HFOs” due to such artifacts, but more effort is needed to analyze awake EEGs. Second, the sample size in the present study was small (*n* = 50 in total). A larger sample size would have allowed for multivariate analysis with variables such as age and medication effects as adjustment factors. Third, this study was conducted on a population of patients receiving antiepileptic drugs. Therefore, depending on the status of the antiepileptic medication, the results may not necessarily be consistent. However, there is also evidence from animal models that seizure suppression is reflected in a reduction in the HFO detection rates, independently of the status of the antiepileptic medication [48]. Fourth, the EEG data used for the analysis in this study were recorded at a sampling frequency of 1000 Hz. In general, equipment that records at a sampling rate at least three times higher than the upper-frequency limit of interest is required. Therefore, most but not all of the scalp HFOs in the 80–250 Hz frequency range were probably detected. Future validation of the study results with higher sampling frequencies is needed.

## 5. Conclusions

The results of the present study indicated that scalp HFOs were more common and had a higher detection rate in patients with BASED scores of 4 and 5. Furthermore, an increased risk of a high scalp HFO detection rate was observed in patients with a BASED score of 5 compared with a BASED score ≤ 2. Finally, scalp HFO detection rates showed better performance in estimating patients with BASED scores of 4 and 5. At present, scalp HFOs cannot be directly applied to guide the diagnosis and treatment of IESS, nor can their reliability be completely determined. In the future, larger prospective longitudinal studies could help establish a robust platform to utilize both BASED scores and scalp HFOs as clinically useful biomarkers for IESS.

## Figures and Tables

**Table 1:** Basic characteristics of the study participants.

	Patients Without Epilepsy (*n* = 25)	Patients with IESS (*n* = 25)	*p*
Sex (*n*, %)			
Male	11 (44.0)	13 (52.0)	0.57 ^‡^
Female	14 (56.0)	12 (48.0)	
Age at the time of EEG recording (years) *	3.7 ± 4.6	4.5 ± 4.5	0.54c ^§^
Age at IESS onset (years) *	–	0.5 ± 1.3	–
IESS duration (day) *	–	1345.6 ± 1365.0	–
Medication (*n*, %)			
VPA	–	11 (44.0)	–
VGB	–	7 (28.0)	
ZNS	–	7 (28.0)	
LEV	–	2 (8.0)	
PER	–	2 (8.0)	
TPM	–	1 (4.0)	
Others	–	7 (28.0)	
Two drugs combined	–	6 (24.0)	
More than two drugs combined	–	7 (28.0)	
ACTH therapy history (*n*, %)	–	10 (40.0)	–
Sleep-inducing agent user during EEG recording (*n*, %)	14 (56.0)	6 (24.0)	0.02 ^‡^
Analyzed EEG recording duration (min) ^†^	600.0 (551.5–601.0)	600.0 (536.5–601.0)	0.59 ^§^
BASED score (*n*, %)			
0	21 (84.0)	0 (0.0)	<0.001 ^‡^
1	4 (16.0)	0 (0.0)	
2	0 (0.0)	1 (4.0)	
3	0 (0.0)	6 (24.0)	
4	0 (0.0)	8 (32.0)	
5	0 (0.0)	10 (40.0)	

Abbreviations: ACTH, adrenocorticotropic hormone; BASED score, burden of amplitudes and epileptiform discharges score; EEG, electroencephalography; IESS, infantile epileptic spasms syndrome; TPM, topiramate; VGB, vigabatrin; VPA, valproic acid. * Data are expressed as mean ± SD. ^†^ Data are expressed as geometric mean (25th–75th percentiles). ^‡^ χ^2^ test. ^§^ Student’s *t*-test.

**Table 2:** Comparison of scalp HFOs in patients with IESS with good and poor seizure control and patients without epilepsy.

	Patients Without Epilepsy (*n* = 25)	Patients with IESS (*n* = 25)		*p*
		Good Seizure Control (*n* = 7)	Poor Seizure Control (*n* = 18)	
Scalp HFO appearance (*n*, %)	2 (8.0)	18 (72.0)		<0.001 ^†^
Scalp HFO detection rates (detections/min) *	0.00 (0.00–0.00)	1.04 (0.00–4.45)		<0.001 ^‡^
Scalp HFO appearance (*n*, %)	2 (8.0)	3 (42.9)	15 (83.3)	<0.001 ^†^
Scalp HFO detection rates (detections/min) *	0.00 (0.00–0.00)	0.00 (0.00–0.30) ^||^	1.77 (0.60–6.36) ^¶,#^	<0.001 ^§^

Abbreviations: HFO, high-frequency oscillation; IESS, infantile epileptic spasms syndrome. * Data are expressed as geometric mean (25th–75th percentiles). ^†^ χ^2^ test. ^‡^ Wilcoxon rank-sum test. ^§^ Kruskal–Wallis rank sum test. ^||^ *p* < 0.05 (vs. patients without epilepsy; Steel–Dwass multiple comparison test). ^¶^ *p* < 0.001 (vs. patients without epilepsy; Steel–Dwass multiple comparison test). ^#^ *p* < 0.01 (vs. good seizure control; Steel–Dwass multiple comparison test).

**Table 3:** Odds ratios and 95% confidence intervals of scalp HFOs according to BASED scores.

BASED Score	Proportion (%)	OR	95% CI	*p*
Scalp HFO appearance				
≤2	7.7%	1.0		
3	16.7%	2.7	0.2–24.7	0.40
4	87.5%	49.0	7.0–652.1	<0.001
5	100%	205.8	18.0–29,736.5	<0.001
Continuous *	40%	3.1	1.9–5.9	<0.001
High scalp HFO detection rate				
≤2	0.0%	1.0		
3	0.0%	4.1	0.02–788.9	0.51
4	12.5%	10.6	0.5–1614.8	0.12
5	90.0%	335.7	25.0–52,662.8	<0.001
Continuous *	20%	65.8	6.5–2562.9	0.004

Abbreviations: BASED score, burden of amplitudes and epileptiform discharges score; CI, confidence interval; HFO, high-frequency oscillations; OR, odds ratio. * Odds ratios were calculated when the BASED score was 1 higher.

## Data Availability

The data presented in this study are available on request from the corresponding author.

## Supplementary Materials

The following supporting information can be downloaded at: https://www.mdpi.com/article/10.3390/biom15050697/s1, Table S1: The 2021 BASED score.

## Abbreviations

The following abbreviations are used in this manuscript:

AUC-ROC	Area under the ROC curve
BASED	Burden of AmplitudeS and Epileptiform Discharges
EEG	Electroencephalography
CI	Confidence interval
HFO	High-frequency oscillation
IESS	Infantile epileptic spasms syndrome
IQRs	Interquartile ranges
IRR	Inter-rater reliability
OR	Odds ratio
ROC	Receiver operating characteristic
Scalp HFO	HFOs measured with scalp EEG
SD	Standard deviation

## References

[1] Specchio N., Wirrell E.C., Scheffer I.E., Nabbout R., Riney K., Samia P., Guerreiro M., Gwer S., Zuberi S.M., Wilmshurst J.M. (2022). International League Against Epilepsy classification and definition of epilepsy syndromes with onset in childhood: Position paper by the ILAE Task Force on Nosology and Definitions. Epilepsia.

[2] Ng A.C.-H., Choudhary A., Barrett K.T., Gavrilovici C., Scantlebury M.H. (2024). Mechanisms of infantile epileptic spasms syndrome: What have we learned from animal models?. Epilepsia.

[3] Snyder H.E., Jain P., RamachandranNair R., Jones K.C., Whitney R. (2024). Genetic Advancements in Infantile Epileptic Spasms Syndrome and Opportunities for Precision Medicine. Genes.

[4] Pavone P., Polizzi A., Marino S.D., Corsello G., Falsaperla R., Marino S., Ruggieri M. (2020). West syndrome: A comprehensive review. Neurol. Sci..

[5] Zuberi S.M., Wirrell E., Yozawitz E., Wilmshurst J.M., Specchio N., Riney K., Pressler R., Auvin S., Samia P., Hirsch E. (2022). ILAE classification and definition of epilepsy syndromes with onset in neonates and infants: Position statement by the ILAE Task Force on Nosology and Definitions. Epilepsia.

[6] Fukuyama Y. (2001). History of clinical identification of West syndrome--in quest after the classic. Brain Dev..

[7] Hollenshead P.P., Jackson C.N., Cross J.V., Witten T.E., Anwar A.I., Ahmadzadeh S., Shekoohi S., Kaye A.D. (2024). Treatment modalities for infantile spasms: Current considerations and evolving strategies in clinical practice. Neurol. Sci..

[8] Mytinger J.R., Hussain S.A., Islam M.P., Millichap J.J., Patel A.D., Ryan N.R., Twanow J.-D.E., Heyer G.L. (2015). Improving the inter-rater agreement of hypsarrhythmia using a simplified EEG grading scale for children with infantile spasms. Epilepsy Res..

[9] Mytinger J.R., Vidaurre J., Moore-Clingenpeel M., Stanek J.R., Albert D.V.F. (2021). A reliable interictal EEG grading scale for children with infantile spasms—The 2021 BASED score. Epilepsy Res..

[10] Romero Milà B., Remakanthakurup Sindhu K., Mytinger J.R., Shrey D.W., Lopour B.A. (2022). EEG biomarkers for the diagnosis and treatment of infantile spasms. Front. Neurol..

[11] Wan L., Lei Y.-Q., Liu X.-T., Chen J., Yeh C.-H., Zhang C.-T., Wang X.-A., Shi X.-Y., Wang J., Zhang B. (2022). Assessing Risk for Relapse among Children with Infantile Spasms Using the Based Score after ACTH Treatment: A Retrospective Study. Neurol. Ther..

[12] Mytinger J.R., Albert D.V.F., Aylward S.C., Beatty C.W., Bhalla S., Bhatia S., Brock G.N., Ciliberto M.A., Choudhari P.R., Clark D.J. (2025). A Multicenter Training and Interrater Reliability Study of the BASED Score for Infantile Epileptic Spasms Syndrome. J. Clin. Neurophysiol..

[13] Frauscher B., Bartolomei F., Kobayashi K., Cimbalnik J., van ’t Klooster M.A., Rampp S., Otsubo H., Höller Y., Wu J.Y., Asano E. (2017). High-frequency oscillations: The state of clinical research. Epilepsia.

[14] Maeda K., Hosoda N., Fukumoto J., Kawai S., Hayafuji M., Tsuboi H., Fujita S., Ichino N., Osakabe K., Sugimoto K. (2025). Association of Scalp High-Frequency Oscillation Detection and Characteristics With Disease Activity in Pediatric Epilepsy. J. Clin. Neurophysiol..

[15] Maeda K., Tsuboi H., Hosoda N., Fukumoto J., Fujita S., Ichino N., Osakabe K., Sugimoto K., Furukawa G., Ishihara N. (2025). Mitochondrial myopathy, encephalopathy, lactic acidosis, and stroke-like episodes (MELAS) with high-frequency oscillations on scalp EEG: A case report. Epilepsy Behav. Rep..

[16] Sueri C., Gasparini S., Balestrini S., Labate A., Gambardella A., Russo E., Leo A., Casarotto S., Pittau F., Trimboli M. (2018). Diagnostic Biomarkers of Epilepsy. Curr. Pharm. Biotechnol..

[17] Menendez de la Prida L., Gotman J. (2024). High-Frequency Oscillations. Jasper’s Basic Mechanisms of the Epilepsies.

[18] Remakanthakurup Sindhu K., Staba R., Lopour B.A. (2020). Trends in the use of automated algorithms for the detection of high-frequency oscillations associated with human epilepsy. Epilepsia.

[19] Kobayashi K., Endoh F., Agari T., Akiyama T., Akiyama M., Hayashi Y., Shibata T., Hanaoka Y., Oka M., Yoshinaga H. (2017). Complex observation of scalp fast (40–150 Hz) oscillations in West syndrome and related disorders with structural brain pathology. Epilepsia Open.

[20] Nariai H., Beal J., Galanopoulou A.S., Mowrey W.B., Bickel S., Sogawa Y., Jehle R., Shinnar S., Moshé S.L. (2017). Scalp EEG Ictal gamma and beta activity during infantile spasms: Evidence of focality. Epilepsia.

[21] Iwatani Y., Kagitani-Shimono K., Tominaga K., Okinaga T., Kishima H., Kato A., Nagai T., Ozono K. (2012). Ictal high-frequency oscillations on scalp EEG recordings in symptomatic West syndrome. Epilepsy Res..

[22] Nariai H., Nagasawa T., Juhász C., Sood S., Chugani H.T., Asano E. (2011). Statistical mapping of ictal high-frequency oscillations in epileptic spasms. Epilepsia.

[23] Noorlag L., van Klink N.E.C., Kobayashi K., Gotman J., Braun K.P.J., Zijlmans M. (2022). High-frequency oscillations in scalp EEG: A systematic review of methodological choices and clinical findings. Clin. Neurophysiol..

[24] Wong S.M., Arski O.N., Workewych A.M., Donner E., Ochi A., Otsubo H., Snead O.C., Ibrahim G.M. (2021). Detection of high-frequency oscillations in electroencephalography: A scoping review and an adaptable open-source framework. Seizure.

[25] World Medical Association (2013). World Medical Association Declaration of Helsinki: Ethical principles for medical research involving human subjects. JAMA.

[26] Worrell G.A., Jerbi K., Kobayashi K., Lina J.M., Zelmann R., Le Van Quyen M. (2012). Recording and analysis techniques for high-frequency oscillations. Prog. Neurobiol..

[27] Crépon B., Navarro V., Hasboun D., Clemenceau S., Martinerie J., Baulac M., Adam C., Le Van Quyen M. (2010). Mapping interictal oscillations greater than 200 Hz recorded with intracranial macroelectrodes in human epilepsy. Brain.

[28] Zhou G., Noto T., Sharma A., Yang Q., González Otárula K.A., Tate M., Templer J.W., Lane G., Zelano C. (2021). HFOApp: A MATLAB Graphical User Interface for High-Frequency Oscillation Marking. ENeuro.

[29] Wirrell E., Tinuper P., Perucca E., Moshé S.L. (2022). Introduction to the epilepsy syndrome papers. Epilepsia.

[30] Wirrell E.C., Nabbout R., Scheffer I.E., Alsaadi T., Bogacz A., French J.A., Hirsch E., Jain S., Kaneko S., Riney K. (2022). Methodology for classification and definition of epilepsy syndromes with list of syndromes: Report of the ILAE Task Force on Nosology and Definitions. Epilepsia.

[31] Fan Y., Dong L., Liu X., Wang H., Liu Y. (2021). Recent advances in the noninvasive detection of high-frequency oscillations in the human brain. Rev. Neurosci..

[32] Kobayashi K., Akiyama T., Oka M., Endoh F., Yoshinaga H. (2015). A storm of fast (40–150Hz) oscillations during hypsarrhythmia in West syndrome. Ann. Neurol..

[33] Kobayashi K., Akiyama T., Oka M., Endoh F., Yoshinaga H. (2016). Fast (40–150Hz) oscillations are associated with positive slow waves in the ictal EEGs of epileptic spasms in West syndrome. Brain Dev..

[34] Zijlmans M., Jacobs J., Zelmann R., Dubeau F., Gotman J. (2009). High frequency oscillations and seizure frequency in patients with focal epilepsy. Epilepsy Res..

[35] Kramer M.A., Ostrowski L.M., Song D.Y., Thorn E.L., Stoyell S.M., Parnes M., Chinappen D., Xiao G., Eden U.T., Staley K.J. (2019). Scalp recorded spike ripples predict seizure risk in childhood epilepsy better than spikes. Brain.

[36] Maeda K., Hosoda N., Tsuboi H., Naito H., Kudo C., Fukumoto J., Fujita S., Ichino N., Osakabe K., Sugimoto K. (2025). The appearance of scalp high-frequency oscillations is associated with poor seizure control in pediatric epilepsy patients. Epilepsia Open.

[37] Maeda K., Hosoda N., Fukumoto J., Tsuboi H., Naitou H., Kudou C., Hannya T., Fujita S., Ichino N., Osakabe K. (2025). Relationship between scalp high-frequency oscillations and time since the last seizure in epilepsy. Clin. Neurophysiol..

[38] Jiruska P., Alvarado-Rojas C., Schevon C.A., Staba R., Stacey W., Wendling F., Avoli M. (2017). Update on the mechanisms and roles of high-frequency oscillations in seizures and epileptic disorders. Epilepsia.

[39] Fan Y., Chen D., Wang H., Pan Y., Peng X., Liu X., Liu Y. (2022). Automatic BASED scoring on scalp EEG in children with infantile spasms using convolutional neural network. Front. Mol. Biosci..

[40] Mytinger J.R. (2021). Definitions and Diagnostic Criteria for Infantile Spasms and West Syndrome—Historical Perspectives and Practical Considerations. Semin. Pediatr. Neurol..

[41] Nariai H., Hussain S.A., Bernardo D., Motoi H., Sonoda M., Kuroda N., Asano E., Nguyen J.C., Elashoff D., Sankar R. (2020). Scalp EEG interictal high frequency oscillations as an objective biomarker of infantile spasms. Clin. Neurophysiol..

[42] Tsuchiya H., Endoh F., Akiyama T., Matsuhashi M., Kobayashi K. (2020). Longitudinal correspondence of epilepsy and scalp EEG fast (40–200 Hz) oscillations in pediatric patients with tuberous sclerosis complex. Brain Dev..

[43] Wang W., Li H., Yan J., Zhang H., Li X., Zheng S., Wang J., Xing Y., Cheng L., Li D. (2021). Automatic detection of interictal ripples on scalp EEG to evaluate the effect and prognosis of ACTH therapy in patients with infantile spasms. Epilepsia.

[44] Cserpan D., Guidi G., Alessandri B., Fedele T., Rüegger A., Pisani F., Sarnthein J., Ramantani G. (2023). Scalp high-frequency oscillations differentiate neonates with seizures from healthy neonates. Epilepsia Open.

[45] Nariai H., Wu J.Y., Bernardo D., Fallah A., Sankar R., Hussain S.A. (2018). Interrater reliability in visual identification of interictal high-frequency oscillations on electrocorticography and scalp EEG. Epilepsia Open.

[46] Spring A.M., Pittman D.J., Aghakhani Y., Jirsch J., Pillay N., Bello-Espinosa L.E., Josephson C., Federico P. (2017). Interrater reliability of visually evaluated high frequency oscillations. Clin. Neurophysiol..

[47] Bénar C.G., Chauvière L., Bartolomei F., Wendling F. (2010). Pitfalls of high-pass filtering for detecting epileptic oscillations: A technical note on “false” ripples. Clin. Neurophysiol..

[48] Lévesque M., Behr C., Avoli M. (2015). The anti-ictogenic effects of levetiracetam are mirrored by interictal spiking and high-frequency oscillation changes in a model of temporal lobe epilepsy. Seizure.

