# Supplementary material for: Association Between Scalp High-Frequency Oscillations and Burden of Amplitudes and Epileptiform Discharges (BASED) Scores in Infantile Epileptic Spasms Syndrome
Source: Biomolecules. 2025 May 10;15(5):697. doi: 10.3390/biom15050697 (PMC12108796; doi:10.3390/biom15050697)
Supplement: Supplementary file 1 [file biomolecules-15-00697-s001.zip › Supplemental Table S1.pdf]

Supplemental Table S1. The 2021 BASED score.

BASED score	Description
**0**	Normal
**1**	Any definite non-epileptiform abnormality
**2**	<3 spike foci **AND** no channel with abnormal high amplitude
**3**	>3 spike foci <50% of one-second bins **AND** no channel with abnormal high amplitude, **OR**<br><3 spike foci but >1 channel with abnormal high amplitude
**4 (Probable EE)**	≥3 spike foci <50% of one-second bins **AND** ≥1 channel with abnormal high amplitude, **OR**<br>Not meeting criteria for 5 but includes GMFS or paroxysmal voltage attenuations
**5 (Definite EE)**	≥3 spike foci that are ≥50% of one second bins

Abbreviations: BASED: Burden of AmplitudeS and Epileptiform Discharges, GMFS: grouped multifocal spikes, EE: epileptic encephalopathy.

BASED Score Rules: Apply score 3–5 to the most epileptic 5 min epoch; if no score is reached, apply score 0–2 to the remainder of the study

>3 Spike Foci Rules:

1. Maybe at least one from each hemisphere OR all from one hemisphere (may include midline)
2. If >3 spike foci in the entire study but no 3 spike foci within 5 min, and no channel with abnormally high amplitude, the BASED score is 2 + uncommon multifocal spikes

Spike Burden Rules:

1. % one-second bins that include 1 or more spikes in the most epileptic 5 min epoch
2. Calculate >50 % >3 spike foci by determining if 10 or more 15 s pages in a 5 min epoch include >8/15 one-second bins with a spike

Amplitude Rules:

1. Peak-to-peak amplitude on a longitudinal bipolar montage, refers to background waves and excludes 1) the slow wave of a preceding spike and the field of these waves in other channels, 2) hypnagogic patterns, and 3) arousal rhythms
2. Waves must be common: present at least once in 10 or more 15 s pages in a 5 min epoch
3. Abnormal high amplitude:

a. >200 µv: Fp1-F7, F7-T3, Fp1-F3, F3-C3, C3-P3, Fp2-F4, F4-C4, C4-P4, C4-P4, Fp2-F8, F8-T4

b. >300 µv: T3-T5, T4-T6

c. Excluded: Fz-Cz, Cz-Pz, T5-O1, P3-O1, P4-O2, T6-O2

Grouped Multifocal Spikes Definition:

1. At least 2 different spike foci in each hemisphere within a fairly well-delineated group (may include midline)
2. For hemispheric grouping, at least 3 different spike foci in one hemisphere (may include midline) within a fairly well-delineated group

Paroxysmal Voltage Attenuation Definition:

1. Definite sudden change from ongoing background activities appearing as a relative attenuation most often lasting one second (but may last several seconds), often occurring after an epileptic discharge, and usually diffuse (but may be present in only one hemisphere)

Remission Rules:

1. Pretreatment score of 4 or 5, must improve to <3
2. Pretreatment score of 3, must improve to <2
